# Oncologic Outcomes of Renal Cell Carcinoma Patients Undergoing Radical Nephrectomy and Venous Thrombectomy: Prospective Follow-Up from a Single Center

**DOI:** 10.1155/2022/9191659

**Published:** 2022-03-17

**Authors:** Yu Zhang, XiaoJun Tian, Hai Bi, Peng Hong, Zhuo Liu, Ye Yan, Cheng Liu, LuLin Ma

**Affiliations:** Department of Urology, Peking University Third Hospital, Beijing, China

## Abstract

**Purpose:**

To evaluate the long-term oncologic outcomes of renal cell carcinoma (RCC) patients with venous thrombus after radical nephrectomy and venous thrombectomy (RN-VT) and to determine the prognostic factors.

**Methods and Materials:**

We reported our follow-up data of RCC patients with venous thrombus from January 2014 to September 2020. We used the Kaplan-Meier method to assess the overall survival (OS), cancer-specific survival (CSS), and recurrence-free survival (RFS). The Cox proportional hazards regression model and competing risk model were used.

**Results:**

After a median follow-up of 31 mon, eight-five patients (31.5%) died, and cancer-specific deaths occurred in 60 patients (22.2%). The 1 yr and 3 yr CSS were 89.3% and 72.7%, respectively. The median OS was 56.0 mon (95% CI 47.6-64.3 mon), and the 1 yr, 3 yr, and 5 yr OS were 87.0%, 62.1%, and 44.8%, respectively. For M1 patients, the median OS was 27.0 mon (95% CI 22.0-42.0 mon), and the 1 yr, 3 yr, and 5 yr OS were 78.0%, 41.5%, and 23.3%, respectively. For M0 patients, the median RFS was 38.0 mon (95% CI 32.5-43.5 mon), and the 1 yr and 3 yr RFS were 81.2% and 52.3%, respectively. Multivariate analyses showed that papillary RCC (HR 2.95, 95% CI 1.80-4.82, *P* < 0.001) or other RCC (HR 3.88, 95% CI 2.03-7.41, *P* < 0.001), perinephric fat invasion (HR 1.53, 95% CI 1.03-2.26, *P* = 0.04), sarcomatoid differentiation (HR 2.85, 95% CI 1.64-4.95, *P* < 0.001), Fuhrman grade 3 (HR 2.10, 95% CI 1.28-3.44, *P* = 0.003) or 4 (HR 3.55, 95% CI 2.09-6.03, *P* < 0.001), and distant metastasis (HR 1.76, 95% CI 1.18-2.63, *P* = 0.006) were associated with a worse CSS. Adjuvant therapy (HR 0.63, 95% CI 0.43-0.92, *P* = 0.02) was associated with a better CSS.

**Conclusions:**

RCC patients can have an acceptable long-term survival after RN-VT. Prognostic factors influencing CSS included nonclear cell RCC histology, higher Fuhrman grade, sarcomatoid differentiation, perinephric fat invasion, distant metastasis, and adjuvant therapy.

## 1. Introduction

Renal cell carcinoma (RCC) can invade the venous system and approximate 4% to 10% RCC patients that had venous thrombus [1]. Radical nephrectomy and venous thrombectomy (RN-VT) can offer reasonable long-term survival, and the 5 yr cancer-specific survival (CSS) rate can be 46% [2–4]. The impact of thrombus level on the survival of such patients has been analyzed, and the conclusions were controversial. Some studies [2, 5] found that thrombus level did not have an explicit association with CSS while a multicenter study [6] showed that thrombus level was an independent prognostic factor. A critical evaluation of prognostic factors on RCC patients with venous thrombus can provide insight into the potential oncologic benefit after RN-VT.

In this study, we prospectively evaluated the long-term survival in RCC patients with venous thrombus to report the oncologic outcomes of such patients in China and to determine the prognostic factors. We hypothesized that patients could have acceptable oncologic outcomes after RN-VT, and the thrombus level was a prognostic factor.

## 2. Materials and Methods

### 2.1. Study Cohort

We have been building the Thrombus Database (TD) since January 2014 after obtaining the institutional review board approval. In this study, we reported the follow-up data of patients from January 2014 to September 2020 (*n* = 270), and all patients were followed up to March 2021. The inclusive criteria were as follows: (1) patients with pathologically confirmed RCC and (2) patients treated with surgical procedures. The patients with a minimum follow-up of less than 6 mon were excluded.

### 2.2. Surgical Procedures

We have described both laparoscopic procedure and open procedure when performing RN-VT at our institution [7–9]. The surgical decision was made based on the physical status, surgery history, thrombus level, and involvement of vein wall assessed by preoperative abdominal magnetic resonance imaging (MRI).

### 2.3. Follow-Up

Two full-time clinical data managers had all access to the database and performed the follow-up. Follow-up data were prospectively collected (symptoms and signs, laboratory tests, imaging examination of the chest, abdomen and pelvis). The laboratory tests included routine blood test and blood biochemical test. The imaging examination included computed tomography (CT), MRI, and X-ray. Patients were followed up every 3 mon after surgery in the first year, then 6 mon to the third year, and then annually thereafter. Except for the routine review after surgery, the data managers conducted telephone interviews every 3-6 mon and collected the follow-up information to reduce the withdraw bias.

### 2.4. Outcomes and Definitions

The primary outcome was the CSS after surgery. Besides that, we also assessed the overall survival (OS) and the recurrence-free survival (RFS). CSS was defined as the time from surgery to death due to cancer. Perioperative mortality (death within 30 d of surgery) was analyzed for CSS. OS meant the length of time from surgery to death from any cause. RFS was defined as the time from surgery to local recurrence (tumor recurrence in or abutting the previous surgical bed) or to the development of metastatic disease (new lesions in other organs, brain, lung, liver, bone, etc. based on CT or MRI). RFS was only assessed in patients with M0 diseases. We determined the death reason according to the death certificate issued by the local medical institution if a patient died during the interval of follow-up visit or we determined that by the treating physicians.

Local symptoms were defined as a palpable mass, pain, and gross hematuria. Patients with edema, fever, swelling, fatigue and weight loss, etc. were thought to have systemic symptoms. The American Society of Anesthesiologists Physical Status Classification System (ASA level) [10] was introduced to estimate the operative risk. Complications were graded according to the Clavien-Dindo grading system [11].

We classified the thrombus into three levels: (1) level I, renal vein thrombus (Mayo 0); (2) level II, thrombus extending the renal vein but below the intrahepatic vena cava (Mayo I and II), and (3) level III, thrombus extending into the intrahepatic vena cava or even into the right atrium (Mayo III and IV) [1, 5]. The histological diagnosis of renal tumors was based on the World Health Organization (WHO) classification (2004 and 2016 version) [12, 13]. The Fuhrman system was applied to RCC nuclear grading [14]. A sarcomatoid differentiation was defined as RCC accompanied by histological appearance of spindle-cell sarcoma. The 2017 version of the tumor-node-metastasis (TNM) classification was used for clinical staging based on postoperative pathological specimen.

### 2.5. Statistical Analysis

Baseline characteristics were shown for categorical variables and continuous variables. Nonnormally distributed continuous variables were reported as medians and interquartile ranges, and normally distributed continuous variables were reported as means and standard deviations. We reported the categorical variables as frequencies and proportions. The chained multiple imputation was used to resolve the missing data.

We used the Kaplan-Meier method to perform survival analysis. Cox proportional hazards regression model was used to estimate the CSS and OS after adjusting the variables that satisfied the proportional hazards assumptions. A competing risk model was also generated for RFS analysis with death as the competing event.

All statistical tests were performed by SPSS version 25.0 (IBM, Armonk, NY, USA) and the *R* statistics package version 4.1.0 (R Project for Statistical Computing, http://www.r-project.org). All tests were two-sided, and the significance level was set at *P* < 0.05.

## 3. Results

### 3.1. Baseline Characteristics of Study Cohort

Baseline characteristics were summarized in Table 1. A total of 270 patients with RCC and venous thrombus formed the study cohort, including 67 patients (24.8%) with level I thrombus, 153 patients (56.6%) with level II thrombus, and 50 patients (18.6%) with level III thrombus. Among them, 82 patients (30.4%) had distant metastasis presentation in diagnosis, 25 patients (9.3%) had suspected adrenal metastasis, and 169 patients (62.6%) had suspected lymph node metastasis.

### 3.2. Surgical Outcomes and Pathological Characteristics

Laparoscopic surgery was performed in 130 patients (48.1%), and 140 patients (51.9%) received open surgery. Perioperative deaths occurred in 4 patients (1.5%), and 88 patients (32.6%) had complications. Of the 270 patients, 225 patients (83.3%) had clear cell RCC, 30 patients (11.1%) had papillary RCC, and 15 patients (5.6%) had other RCC types, including chromophobe RCC (2 cases, 0.7%), squamous RCC (1 case, 0.4%), TFE-3 translocation RCC (5 cases, 1.9%), and unclassified RCC (7 cases, 2.6%). Lymph node metastasis was confirmed in 93 patients (34.4%), and perinephric fat invasion was found in 73 patients (27.0%). The venous wall was involved in 58 patients (21.5%), and 45 patients received segmental resection of IVC. A total of 155 patients (57.4%) received adjuvant therapy. The clinical and pathological features were shown in Table 2.

### 3.3. Survival Analysis of the Study Cohort

**Figure 1:**
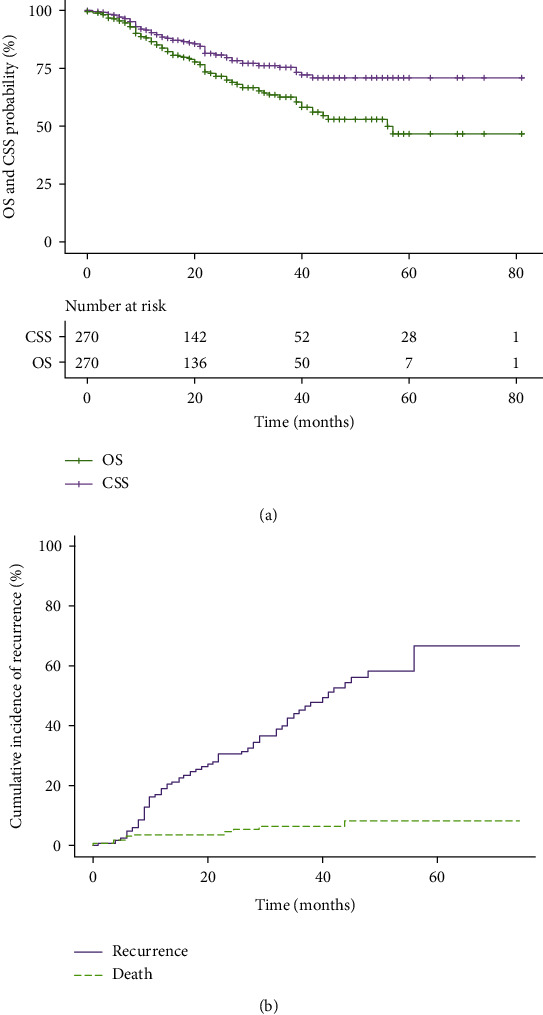
(a) Adjusted CSS and OS of the study cohort. (b) Cumulative incidence of recurrence in M0 patients (*n* = 188).

Table 3 summarized the survival data of CSS, OS, and RFS and presented them in groups. After a median follow-up of 31 mon, eight-five patients (31.5%) died, and cancer-specific deaths occurred in 60 patients (22.2%). The median CSS was not reached, and the 1 yr and 3 yr CSS were 89.3% and 72.7%, respectively. For M1 patients, the 1 yr and 3 yr CSS were 83.2% and 55.6%, respectively, lower than 90.0% and 75.3% of M0 patients. Variables associated with CSS were shown in Table 4. Although the thrombus level showed no association with CSS in univariate analysis, it was still included in the multivariate analysis from a clinical point of view. However, only the Fuhrman grade (grade 3 HR 2.10, 95% CI 1.28-3.44; *P* = 0.003, grade 4 HR 3.55, 95% CI 2.09-6.03; *P* < 0.001), pathological type (papillary RCC HR 2.95, 95% CI 1.80-4.82; *P* < 0.001, other RCC (HR 3.88, 95% CI 2.03-7.41; *P* < 0.001), perinephric fat invasion (HR 1.53 95% CI 1.03-2.26; *P* = 0.04), sarcomatoid differentiation (HR 2.85 95% CI 1.64-4.95; *P* < 0.001), and adjuvant therapy (HR 0.63 95% CI 0.43-0.92; *P* = 0.02) were the prognostic factors of CSS according to the multivariate analysis.  Figure 1(a) depicted the CSS curve.

The median OS was 56.0 mon (95% CI 47.6-64.3 mon), and the 1 yr, 3 yr, and 5 yr OS were 87.0%, 62.1%, and 44.8%, respectively. For M1 patients, the median OS was 27.0 mon (95% CI 22.0-42.0 mon), and the 1 yr, 3 yr and 5 yr OS were 78.0%, 41.5%, and 23.3%, respectively. Multivariate analysis showed that Fuhrman grade (grade 3 HR 1.82, 95% CI 1.22-2.72; *P* = 0.003, grade 4 HR 2.92, 95% CI 1.88-4.53; *P* < 0.001), pathological type (papillary RCC HR 2.13, 95% CI 1.38-3.30; *P* = 0.001, other RCC HR 2.67, 95% CI 1.51-4.73; *P* = 0.001), sarcomatoid differentiation (HR 2.55 95% CI 1.60-4.08; *P* < 0.001), and distant metastasis (HR 2.17, 95% CI 1.55-3.04; *P* < 0.001) were associated with OS (Table 4).  Figure 1(a) depicted the OS curve.

For M0 patients (*n* = 188), 68 patients (36.2%) had postoperative recurrence. The median RFS was 38.0 mon (95% CI 32.5-43.5 mon), and the 1 yr and 3 yr RFS were 81.2% and 52.3%, respectively (Table 3). The median RFS for level I, II, and III thrombus was 38.7 mon (95% CI 33.6-54.4 mon), 30.9 mon (95% CI 28.8-37.1 mon), and 23.3 mon (95% CI 18.8-37.2 mon), respectively. The 3 yr RFS for level I, II, and III thrombus was 80.4%, 56.0%, and 35.4%, respectively.  Figure 1(b) showed the cumulative incidence of recurrence.

## 4. Discussion

With the advances of surgical technique and systemic therapy, the long-term survival of RCC patients with venous thrombus is expected to improve. In this study, we evaluated the oncologic outcomes and predictors of survival of surgically treated RCC patients with venous thrombus. Our data indicated that the 3 yr CSS after RN-VT could be 72.7% of the entire cohort and 55.6% of the M1 patients. Though the median CSS was not reached in our study, we found the median OS of the entire cohort was 56 mon and only 27 mon for M1 patients. In our multivariate analysis, we could not observe an association between thrombus level and survival.

Several studies have reported the CSS or OS of RCC patients with venous thrombus. Haferkamp et al. [5] reported that the median CSS in non-N0M0 patients after surgery was 10.7 mon compared with 51.7 mon in N0M0 patients. Haddad et al. [15] reported in a retrospective multicenter study that for M1 patients with thrombus extending above the hepatic veins, the median OS was only 10.7 mon, and all deaths were due to progressive malignancy. Lambert et al. [16] once reported that the 5 yr CSS was 60.3% for M1 patients and only 10% for M1 patients. The 5 yr CSS was not reached in our analysis, but we observed that the 3 yr CSS of pN0-xM0 patients was higher than that of pN1M0 or M1 patients. At our institution, the 5 yr OS was 23.3%, and the median OS was 27.0 mon for M1 patients. Though the radical surgery was confirmed to improve the long-term survival, whether we should perform surgery in M1 patients was still a question and needs to be validated by further prospective study.

Ciancio et al. [2] found that the 5 yr OS was 39% for RCC patients with IVC thrombus. Compared with the study of Ciancio et al., the 5 yr OS in our cohort was higher (44.8% vs. 39%), and the following reasons could explain the discrepancy. Firstly, we included the patients with renal vein involvement into analysis while they reported the OS of patients with only IVC thrombus. Next, the proportion of nuclear grades III and IV was much higher in their cohort (74% vs. 61.9%). Moreover, nonclear cell RCC accounted for 29% of their cohort, and it was confirmed to be associated with poor prognosis. While in our study, the proportion was only 16.7%. In addition, the shorter follow-up in their study (22 mon vs. 31 mon) might be another explanation. Lastly, they performed RN-VT by open approach while the minimally invasive procedures and open approach were used in our center. The advantages of minimally invasive procedures, including laparoscopic approach and robotic approach, might also contribute to the benefit in OS [17]. However, it should be validated in further study.

The recurrence of RCC patients with venous thrombus after surgery was another concern. Our analysis showed that the median RFS was 38.0 mon for all M0 patients.

However, Abel et al. [18] reported the median RFS was only 9.0 mon. One major reason was that only 19 patients (4.1%) received postoperative adjuvant therapy in their study while the proportion was 57.4% in our cohort. Another possible explanation was that the proportion of nuclear grades III and IV was much higher in their cohort (81.1% vs. 61.9%), which could result in a worse RFS. Haddad et al. [15] observed that the median RFS was 15.2 mon for M0 patients with thrombus extending above the hepatic veins, and the 1 yr and 3 yr RFS were 55.7% and 35.9%, respectively. While in our cohort, the median RFS was 23.3 mon for M0 patients with thrombus extending above the hepatic veins, and the 1 yr and 3 yr were 68.7% and 35.4%, respectively. The systemic recurrence rate was also lower in our study (31.9% vs. 49.2%). Actually, we thought that the higher postoperative adjuvant therapy rate (57.4%) might help reduce recurrence or postpone the recurrence time in our cohort. Motzer et al. [19] once reported that adjuvant therapy was a protective factor for patients > 65 yr after nephrectomy but was not related to survival for patients aged 45 yr to 64 yr. Therefore, the exact effect of adjuvant therapy on RFS in RCC patients with venous thrombus and the association between age and survival still need further study to validate.

Identifying the variables influencing the oncologic outcomes is crucial to improve the survival. We found that thrombus level was not associated with the long-term survival of RCC patients, which was consistent with previous studies [2, 15, 16, 20, 21]. Haddad et al. [15] found no difference in CSS in RCC patients with thrombus extending above the hepatic veins, though the median CSS was better in patients with infradiaphragmatic IVC thrombus than patients with supradiaphragmatic IVC thrombus (37.0 mon vs. 20.3 mon). Ciancio et al. [2] observed that patients with thrombus above the diaphragm had less CSS than patients with renal vein thrombus only, and there was no significant difference in survival among other levels. Wagner et al. [20] reported that patients with renal vein thrombus had a significant better OS than patients with IVC thrombus (either above or below the diaphragm) in univariate analysis, but no prognostic value in multivariate analysis. However, Haferkamp et al. [5] observed that thrombus level was a prognostic factor of CSS, and Tilki et al. [6] confirmed that higher thrombus level was associated with reduced CSS. In our study, we found no significant association between thrombus level and survival. It was noteworthy that thrombus level was an independent predictor of RFS in M0 patients according to the study of Abel et al. [18]. Though the prognostic value was still controversial, we thought that thrombus level should be considered when evaluating the risk of recurrence and whether to perform systemic adjuvant therapy.

The impact of pathological type on survival has been evaluated in RCC patients with venous thrombus [6, 15, 20, 22]. Kim et al. [22] reported that patients with type II papillary RCC had significantly lower CSS and RFS than those with clear cell RCC and confirmed that histology was an important prognostic factor. Tilki et al. [6] also confirmed in a multicenter study that patients with papillary RCC and venous thrombus had worse CSS than patients with other subtypes of RCC. Our results were consistent with theirs and the discrepancy of survival among the subtypes of RCC reminded us that we should give closer follow-up to the nonclear cell RCC patients. Abel et al. [18] observed that nonclear cell RCC was also associated with recurrence in M0 patients. The reason of nonclear cell RCC leading to worse survival should be further studied. Actually, when it comes to the prognosis, we should not neglect the role of metabolic signature in ccRCC as described in previous studies [23, 24].

The presence of perinephric fat invasion, Fuhrman grade, and sarcomatoid differentiation were identified as prognostic factors in our study as well as previous studies [2, 6]. However, perinephric fat invasion only influenced CSS but not OS in our analysis. Besides that, patients with adjuvant therapy had a better CSS but not a better OS than patients without adjuvant therapy. We understand this from two perspectives. On the one hand, adjuvant therapy is a beneficial option to improve the CSS of the patients. On the other hand, whether we should give adjuvant therapy to all patients is still questionable due to no significant improvement in OS. Patients with high risk of comorbidity or poor physical status after surgery may not benefit enough from adjuvant therapy.

Our study has strengths in the prospective follow-up design and prospective data collection. In addition, the study period is from January 2014 to March 2021, and it can represent the current clinical practice, especially the comprehensive therapy based on surgical treatment. However, some limitations should be considered. The first is its single-center experience nature, and it contains a relatively small number of patients. A multicenter prospective follow-up covering sufficient data is needed to better evaluate the long-term oncologic outcomes. Furthermore, a relatively shorter follow-up time limited the observation of oncologic outcome events, especially for cancer-specific death. This study would definitely benefit from a longer follow-up.

## 5. Conclusion

In this prospective follow-up cohort, we found that RCC patients could have an acceptable long-term survival after RN-VT. M1 patients had a relatively lower 5 yr OS and 3 yr CSS compared with M0 patients. Patients with nonclear cell RCC histology, higher Fuhrman grade, sarcomatoid differentiation, perinephric fat invasion, distant metastasis, and no adjuvant therapy had a worse CSS. Thrombus level was not associated with survival.

## Figures and Tables

**Table 1:** Baseline characteristics of the study cohort (*n* = 270).

Characteristics	Value
Age (yr), median (IQR)	60 (54-67)
Gender (*n*/%)	
Male	204 (75.6)
Female	66 (24.4)
BMI (kg/m^2^), median (IQR)	23.7 (21.3-26.0)
Laterality (*n*/%)	
Left	101 (37.4)
Right	169 (62.6)
ASA level (*n*/%)	
1	15 (5.6)
2	215 (79.6)
3	37 (13.7)
4	3 (1.1)
Symptoms (*n*/%)	
Local	193 (71.5)
Systemic	77 (28.5)
Comorbidity (*n*/%)	
Hypertension	115 (42.6)
Coronary heart disease	13 (4.8)
Diabetes mellitus	30 (11.1)
Cerebrovascular disease	5 (1.9)
Chronic lung disease	2 (0.7)
Surgery history	67 (24.8)
Preoperative targeted therapy (*n*/%)	13 (4.8)
Tumor diameter (cm) median (IQR)	8.7 (6.6-10.5)
Preoperative SCR (*μ*moL/L), median (IQR)	91 (80.8-110)
Thrombus level (*n*/%)	
I	67 (24.8)
II	153 (56.6)
III	50 (18.6)
Pulmonary embolism (*n*/%)	6 (2.2)
Metastasis at diagnosis (*n*/%)	
Suspected lymph node metastasis	169 (62.6)
Suspected adrenal metastasis	25 (9.3)
Distant metastasis	82 (30.4)
Lung	56 (20.7)
Liver	19 (7.0)
Bone	20 (7.4)

BMI: body mass index; ASA: American Society of Anesthesiologists; SCR: serum creatine; IQR: interquartile range.

**Table 2:** Surgical and pathological outcomes of the study cohort.

Characteristics	Value
Type of surgery	
Laparoscopic surgery	130 (48.1)
Open surgery	140 (51.9)
Adrenalectomy (*n*/%)	123 (45.6)
Segmental resection of IVC (*n*/%)	45 (16.7)
Resection of metastatic tumor (*n*/%)	4 (1.5)
Operative time (min), median (IQR)	320 (240-404)
Blood loss (ml), median (IQR)	700 (200-1800)
Blood transfusion (*n*/%) 1133llchmu109f	136 (50.4)
Packed RBC transfusion (ml), median (IQR)	1200 (800-2000)
FFP transfusion (ml), median (IQR)	600 (400-800)
Postoperative SCR	98 (79-116)
Complications (*n*/%)	88 (32.6)
Cardiovascular or cerebrovascular events	2 (0.7)
Pneumonia or pleural effusion	10 (3.7)
Kidney insufficiency	10 (3.7)
Abdominal cavity infection	4 (1.5)
Incision infection	2 (0.7)
Deep venous thrombus	23 (8.5)
Anemia	22 (8.1)
Bowel obstruction	16 (5.9)
Lymphatic fistula	9 (3.3)
Death	4 (1.5)
Clavien grade of complications (*n*/%)	
I	6 (2.2)
II	51 (18.9)
III	0
IV	11 (4.1)
V	4 (1.5)
Postoperative hospital stay (d), median (IQR)	9 (6-12)
Histology (*n*/%)	
Clear cell RCC	225 (83.3)
Papillary type RCC	30 (11.1)
Chromophobe RCC	2 (0.7)
Unclassified RCC	7 (2.6)
Squamous RCC	1 (0.4)
TFE-3 translocation RCC	5 (1.9)
T stage	
pT3a	50 (8.5)
pT3b	124 (45.9)
pT3c	80 (29.6)
pT4	16 (5.9)
Lymph node metastasis	93 (34.4)
Perinephric fat invasion	73 (27.0)
Involving the venous wall (*n*/%)	58 (21.5)
Sarcomatoid differentiation	30 (11.1)
Fuhrman grade (*n*/%)	
1	5 (1.8)
2	98 (36.3)
3	113 (41.9)
4	54 (20.0)
Adjuvant therapy	155 (57.4%)

RCC: renal cell carcinoma; IVT: inferior vena cava; RBC: red blood cells; FFP: fresh frozen plasma; ASA: American Society of Anesthesiologists; IQR: interquartile range; SCR: serum creatinine.

**Table 3:** Overall survival (OS), cancer-specific survival (CSS), and recurrence-free survival (RFS) of the study cohort.

	OS				CSS^∗^		RFS (*n* = 188)		
	Median mon, (95% CI)	1 yr	3 yr	5 yr	1 yr	3 yr	Median mon, (95% CI)	1 yr	3 yr
All	56.0 (47.6-64.3)	87.0%	62.1%	44.8%	89.3%	72.7%	—	—	—
Distant metastasis									
M1	27.0 (22.0-42.0)	78.0%	41.5%	23.3%	83.2%	55.6%	—	—	—
M0	^∗^	89.4%	68.6%	55.3%	90.0%	75.3%	38.0 (32.5-43.5)	81.2%	52.3%
N0-xM0	^∗^	88.3%	66.2%	51%	89.7%	78.6%	40.7 (34.6-47.4)	84.4%	56.9%
N1M0	^∗^	89.3%	72.6%		91.5%	74.2%	33.2 (28.2-37.7)	70.6%	51.3%
Thrombus level									
I	^∗^	90.5%	78.1%	—	91.5%	78.5%	38.7 (33.6-54.4)	84.7%	80.4%
II	42.0 (34.9-49.1)	87.3%	58.5%	—	89.9%	65.9%	30.9 (28.8-37.1)	85.8%	56.0%
III	40.0 (26.3-44.1)	81.5%	57.9%	36.4%	83.8%	71.6%	23.3 (18.8-37.2)	68.7%	35.4%

OS: overall survival; CSS: cancer-specific survival; RFS: recurrence-free survival; mon: month; CI: confidence interval; ^∗^the median survival was not reached.

**Table 4:** Univariate and multivariate Cox regression analysis of CSS.

	CSS			
Covariate	Univariate analysis		Multivariate analysis	
	HR (95% CI)	*P* value	HR (95% CI)	*P* value
Age	0.98 (0.97-1.01)	0.08		
BMI	0.95 (0.90-0.99)	0.03	0.96 (0.90-1.01)	0.11
Thrombus level (*n*/%)				
I	Reference		Reference	
II	1.44 (0.89-2.34)	0.14	1.27 (0.77-2.10)	0.35
III	1.29 (0.72-2.31)	0.39	0.87 (0.46-1.62)	0.65
Surgery (*n*/%)				
Open procedure	Reference		Reference	
Laparoscopic procedure	0.67 (0.47-0.96)	0.03	0.80 (0.54-1.19)	0.27
Fuhrman grade (*n*/%)				
1-2	Reference		Reference	
3	1.85 (1.16-2.97)	<0.01	2.10 (1.28-3.44)	0.003
4	4.72 (2.93-7.63)	<0.001	3.55 (2.09-6.03)	<0.001
Pathological type (*n*/%)				
Clear cell RCC	Reference		Reference	
Papillary RCC	2.73 (1.73-4.31)	<0.001	2.95 (1.80-4.82)	<0.001
Other	3.63 (2.01-6.53)	<0.001	3.88 (2.03-7.41)	<0.001
Perinephric fat invasion (*n*/%)				
No	Reference		Reference	
Yes	2.14 (1.49-3.08)	<0.001	1.53 (1.03-2.26)	0.04
Sarcomatoid differentiation (*n*/%)				
No	Reference		Reference	
Yes	3.36 (2.18-5.16)	<0.001	2.85 (1.64-4.95)	<0.001
Metastasis (*n*/%)				
N0M0	Reference		Reference	
N1-xM0	0.72 (0.40-1.31)	0.28	0.85 (0.46-1.56)	0.60
M1	1.88 (1.28-2.75)	0.001	1.76 (1.18-2.63)	0.006
Adjuvant therapy (*n*/%)				
No	Reference		Reference	
Yes	0.67 (0.47-0.96)	0.03	0.63 (0.43-0.92)	0.02

CSS: cancer-specific survival; HR: hazard ratio; CI: confidence interval.

## Data Availability

The data can be available to the readers if they e-mail 15010546321@139.com.
